# Observations on the Action of the Broom-Seed in Dropsical Affections

**Published:** 1836-04

**Authors:** 


Art. XIV.—*Observations on the Action of the Broom-Seed in Dropsical Affections.* By Richard Pearson, m.d., late Physician to the Birmingham Hospital, &c.—London. 8vo. 1835. Pp. 15.

In this very diminutive tract Dr. Pearson recommends that the Broom-seed should be substituted for the decoction, as the leaves and root are apt to become mouldy or worm-eaten; whereas the seeds, if gathered when perfectly ripe, and in dry weather, may be kept in stoppered bottles for an indefinite length of time without having their medicinal properties impaired. He recommends the following tincture, the dose of which is one or two drachms three times daily, in water arömatized with tincture of cardamoms, ginger, or cinnamon, and in certain cases three drachms may be given every fourth hour.—℞. Spartii juncei seminum contusorum, ℥ij. Spiritûs tenuioris, ℥viij. Macera per dies decem et cola.

If it produces diarrhœa, five or six drops of tincture of opium may be added to each dose. Due dilution promotes its effect, such as a solution of cream of tartar in water, or, in old gouty subjects, lemon-peal, tea, or weak ginger tea. When dropsy is attended with great debility, this medicine may be combined with quinine and iron. Dr. Pearson considers that the Broom is superior to most other diuretics, and particularly digitalis and squill, from its improving the appetite and invigorating the whole system; whereas the others can only be given for a limited time, as they impair the appetite and digestive powers.

In dropsy depending on visceral disease, Dr. Pearson thinks it is useful in lessening the quantity of fluid, without debilitating the patient, although it cannot remove the disease. He has not tried it in dropsy following scarlatina, but finds it is not adapted to hydrothorax attended with thoracic inflammation, nor to ovarian dropsy. Five cases are given which prove its diuretic action. As it is frequently inconvenient or impracticable to procure the fresh broom-tops, if the substitute which Dr. Pearson has proposed stands the test of farther experience, it will be a useful addition to our diuretic medicines. This little work was Dr. Pearson's last contribution to medicine, of which he was many years ago a distinguished cultivator. His death took place very recently, at a somewhat advanced age.

